# Concordance Between the Multidisciplinary Team and ChatGPT-4o Decisions: A Blinded, Cross-Sectional Concordance Study in Systemic Autoimmune Rheumatic Diseases

**DOI:** 10.3390/diagnostics16010113

**Published:** 2025-12-30

**Authors:** Firdevs Ulutaş, Göksel Altınışık, Gülay Güngör, Vefa Çakmak, Nilüfer Yiğit, Duygu Herek, Murat Yiğit, Uğur Karasu, Veli Çobankara

**Affiliations:** 1 Division of Rheumatology, Department of Internal Medicine, Pamukkale University Faculty of Medicine, 20000 Denizli, Türkiye; 2 Department of Pulmonology, Pamukkale University Faculty of Medicine, 20000 Denizli, Türkiye; 3 Department of Radiology, Pamukkale University Faculty of Medicine, 20000 Denizli, Türkiye

**Keywords:** multidisciplinary team decisions, AI-generated outputs, SARDs, ILD

## Abstract

**Background/Objective:** In recent years, artificial intelligence (AI) has gained increasing prominence in the fields of diagnostic decision-making in medicine. The aim of this study was to compare multidisciplinary team (MDT: rheumatology, pulmonology, thoracic radiology) decisions with single-session plans generated by ChatGPT-4o. **Methods:** In this cross-sectional concordance study, adults (≥18 years) with confirmed systemic autoimmune rheumatic disease (SARD) and having MDT decisions within the last 6 months were included. The study documented diagnostic, treatment, and monitoring decisions in cases of SARDs by recording answers to six essential questions: (1) What is the most likely clinical diagnosis? (2) What is the most likely radiological diagnosis? (3) Is there a need for anti-inflammatory treatment? (4) Is there a need for antifibrotic treatment? (5) Is drug-free follow-up appropriate? and (6) Are additional investigations required? Consequently, all evaluations were performed with ChatGPT-4o in a single-session format using a standardized single-prompt template, with the system blinded to MDT decisions. All data analyses in this study were conducted using the R programming language (version 4.3.2). An agreement between AI-generated and MDT decisions was assessed using Cohen’s Kappa (κ) statistic where κ (kappa) values represent the level of agreement: <0.20 = slight, 0.21–0.40 = fair, 0.41–0.60 = moderate, 0.61–0.80 = substantial, >0.80 = almost perfect agreement. These analyses were performed using the irr and psych packages in R. Statistical significance of the models was evaluated through *p*-values, while overall model fit was assessed using the Likelihood Ratio Test. **Results:** A total of 47 patients were involved in this study, with a predominance of female patients (61.70%, *n* = 29). The mean age was 61.74 ± 10.40 years. The most frequently observed diagnosis was rheumatoid arthritis (RA), accounting for 31.91% of cases (*n* = 15). This was followed by cases of anti-neutrophil cytoplasmic antibody (ANCA)-associated vasculitis, interstitial pneumonia with autoimmune features (IPAF), and sarcoidosis. The analyses indicate a statistically significant level of agreement across all decision types. For clinical diagnosis decisions, agreement was moderate (κ = 0.52), suggesting that the AI system can reach partially consistent conclusions in diagnostic processes. The need for an immunosuppressive treatment and follow-up without medication decisions demonstrated a higher level of concordance, reaching the moderate-to-high range (κ = 0.64 and κ = 0.67, respectively). For antifibrotic treatment decisions, agreement was moderate (κ = 0.49), while radiological diagnosis decisions also fell within the moderate range (κ = 0.55). The lowest agreement—though still moderate—was observed in further investigation required decisions (κ = 0.45). **Conclusions:** In patients with SARDs with pulmonary involvement, particularly in complex cases, concordance was observed between MDT decisions and AI-generated recommendations regarding prioritization of clinical and radiologic diagnoses, treatment selection, suitability for drug-free follow-up, and the need for further diagnostic investigations.

## 1. Introduction

The reported prevalence of systemic autoimmune rheumatic diseases (SARDs) varies across studies, with estimates ranging from 4.5% to 6.4% [1]. SARDs such as systemic lupus erythematosus (SLE), rheumatoid arthritis (RA), Sjögren’s syndrome (SjS), idiopathic inflammatory myopathies (IIM), and systemic sclerosis (SSc) often present with a strikingly diverse spectrum of pulmonary manifestations, including interstitial lung disease (ILD), airway involvement, pleural pathology, diffuse alveolar hemorrhage (DAH), and even discrete pulmonary nodules. Recent mechanistic insights highlight the roles of autophagy dysregulation and post-translational modifications in the pathogenesis, underscoring the molecular complexity underlying SARDs and their heterogeneous clinical behavior [2]. The heterogeneity of these clinical presentations leads to variability in prognoses and treatment choices; even patients with clinically significant and/or progressive disease may warrant immunosuppressive therapy. Identifying which patients truly require such treatment necessitates careful evaluation within a multidisciplinary team (MDT) [3]. In SARDs, ILD represents the most frequent form of pulmonary involvement, although other radiological patterns also occur and are seldom pathognomonic. For instance, rheumatoid or cavitating nodules may closely resemble primary or metastatic lung malignancies, and mucosa-associated lymphoid tissue (MALT) lymphoma may develop in the context of SjS. Moreover, benign interstitial patterns can yield false-positive findings on positron emission tomography (PET). Radiological patterns alone are insufficient for establishing a definitive diagnosis. These challenges highlight the need for radiologists to integrate detailed clinical knowledge in order to reach more accurate radiological diagnoses [4]. In addition to malignancies, infections and drug-induced reactions should also be considered in the differential diagnosis, given the ongoing immunosuppressive treatments [5]. All of these observations underscore the pivotal importance of a multidisciplinary approach involving pulmonologists, rheumatologists, radiologists, and pathologists, enabling early screening, timely intervention, and the selection of the most appropriate treatment to improve patient outcomes. Such collaboration may also help to avoid unnecessary invasive procedures [6]. With the support of the MDT, clinicians can also achieve the early identification of patients with a poor prognosis, thereby facilitating the recognition of progressive disease phenotypes [7]. A MDT approach markedly improves interobserver agreement compared with isolated clinician judgment or single-modality evaluation. Although MDT discussions are resource-intensive, their ability to synthesize complementary clinical, radiological, and pathological expertise underpins their broad acceptance as the reference standard for the diagnosis and management of ILD [8]. Although MDT evaluation is widely regarded as the reference standard for ILD diagnosis, marked heterogeneity in MDT organization and practice across centers continues to compromise diagnostic uniformity. Recently published Delphi consensus recommendations have started to define the core components of an optimal MDT, providing structured guidance on team composition, operational workflows, and shared decision-making frameworks. As a result, the field is moving toward being more standardized and technology-integrated [9].

Chat-generative pretrained transformers-4 (Chat-GPT-4), is an advanced AI language model developed by OpenAI [10]. Chat-GPT-4 is being asserted to have the potential to transform healthcare, medical education, and research, respectively, as a useful learning tool, offering quick access to information and personalized support for students and professionals and help in analyzing large datasets and assisting in developing new methods [11]. In recent years, it has also gained increasing prominence in the fields of diagnostic decision-making [12]. In previous studies, Chat-GPT-4 was compared with emergency physicians and cardiologists in routine electrocardiography (ECG) interpretation and was found to outperform both groups in standard evaluations. In the assessment of complex cases, its diagnostic performance was shown to be comparable to that of cardiologists [13]. Evidence from oncology-based multidisciplinary tumor boards demonstrates that large language models can effectively support clinical teams by retrieving guideline-concordant recommendations, suggesting management options, and structuring follow-up strategies [14].

Although Chat-GPT represents a relatively new AI tool with the potential to support physicians in the diagnosis and management of patients in treatment decisions, its accuracy and reliability in addressing real-world clinical scenarios involving SARDs remain largely unexplored. The aim of this study was to compare MDT decisions with single-session plans generated by Chat-GPT-4o according to the decisions related to diagnostic approaches, treatment strategies, and follow-up decisions in cases of SARDs with pulmonary involvement.

## 2. Materials & Methods

### 2.1. Study Features & Patient Selection

This was a single-center, cross-sectional methodological comparison study. This study received ethical approval from Pamukkale University (Approval number 15; Date: 12 August 2025). The single-center design was deliberately chosen to ensure methodological consistency in MDT composition, diagnostic workflows, and institutional treatment algorithms, thereby minimizing inter-center variability that could confound concordance analyses. All patients (*n* = 47) (≥18 years) with confirmed SARDs and having MDT discussion within the last 6 months in the Division of Rheumatology, at Pamukkale University Hospital, were included in this study. The six-month inclusion window was selected to ensure that all MDT decisions were made within a stable diagnostic and therapeutic framework, thereby minimizing temporal bias related to evolving guidelines, imaging interpretation standards, or institutional treatment strategies. The study documented the MDT decisions, consisting of diagnostic, treatment, and monitoring decisions in SARDs cases by recording answers to six essential questions: (1) What is the most likely clinical diagnosis? (2) What is the most likely radiological diagnosis? (3) Is there a need for anti-inflammatory treatment? (4) Is there a need for antifibrotic treatment? (5) Is drug-free follow-up appropriate? and (6) Are additional investigations required? The outcome of each MDT discussion was documented, including consensus on the most likely clinical and radiological diagnosis, the need for anti-inflammatory or antifibrotic therapy, recommendations for additional diagnostic procedures, and follow-up planning.

The inclusion and exclusion criteria were defined as outlined below.

Inclusion: Adults (≥18 years) with confirmed SARDs (RA, SSc, IIM, SjS, sarcoidosis), available high-resolution computed tomography (HRCT) and pulmonary function tests (PFTs), and having MDT decisions within the last 6 months.

Exclusion: Insufficient data; investigational-drug cases precluding standard plans.

### 2.2. Case Presentation to GBT and Agreement Assessment

#### 2.2.1. Standardized AI Prompt (Full Text Applied Uniformly Across All Cases)

The following fixed prompt was used identically for every interaction:

“You are an expert clinical decision-support system specialized in pulmonary involvement and SARDs. You will receive a de-identified patient vignette containing structured clinical, serological, radiological, and—when available—histopathological data. Your task is to generate a standardized three-part output:

(1) The most likely clinical and radiological diagnosis;
(2) The recommended management plan, including immunosuppressive and/or antifibrotic strategies; and
(3) The need for any additional diagnostic procedures (e.g., detailed HRCT review, bronchoscopy, lung biopsy).

You must base your reasoning strictly on the information provided, without inferring unreported data. Do not introduce external assumptions or probabilistic speculation beyond established evidence-based principles. Use concise terminology aligned with current ATS/ERS and ACR guidelines. After delivering your final recommendation, do not offer alternative scenarios or discuss uncertainty unless explicitly prompted by the vignette. Please review the following patient case and provide your response in the standardized three-section format (‘Diagnosis’, ‘Management’, ‘Further Work-up’).”

#### 2.2.2. Implementation of the Prompt in the Study

All AI-based recommendations were generated using GPT-4o (OpenAI; release current as of May 2025). The model operated in a fully offline environment, without access to web browsing, external databases, proprietary software, or patient-identifiable information. A single, fixed prompt was applied uniformly across all cases, with the temperature set between 0 and 0.2 to maximize output consistency. Each clinical vignette was processed independently within a single-session workflow, precluding any carry-over memory between cases. The AI system remained fully blinded to MDT conclusions. All prompts and outputs were time-stamped and securely archived to ensure full traceability. Human involvement was strictly technical, limited to vignette submission and output retrieval, without any clinical judgment or interpretive input.

Each vignette followed the structure used in MDT deliberations and included patient demographics, presenting symptoms, HRCT characteristics, pulmonary function parameters, such as Forced Vital Capacity (FVC) and Diffusing Capacity of the Lung for Carbon Monoxide (DLCO), serological markers relevant to SARDs, comorbid conditions, and current treatments. Each case summary systematically incorporated features supporting or excluding infectious etiologies, prior treatment history, and documented adverse events to ensure uniform and comprehensive case characterization. Baseline HRCT scans were independently reviewed and coded using DICOM-based classification for established patterns—including nonspecific interstitial pneumonia (NSIP), usual interstitial pneumonia (UIP), organizing pneumonia (OP), lymphocytic interstitial pneumonia (LIP), and diffuse alveolar hemorrhage (DAH)—together with disease extent and key ancillary findings such as traction bronchiectasis, honeycombing, and consolidation [15]. A thoracic radiologist, blinded to all other clinical and MDT data, did not participate in MDT deliberations or outcome scoring, but served exclusively to standardize pattern classification and the corresponding differential diagnosis.

The MDT in our study consisted of four radiologists, four rheumatologists, and two pulmonologists. The MDT operated according to internationally recognized diagnostic principles outlined in the American Thoracic Society (ATS)/European Respiratory Society (ERS) ILD guidelines and American College of Rheumatology (ACR) recommendations for rheumatic disease-associated ILD, thereby ensuring that its consensus decisions reflect current gold-standard methodology [16].

The AI-generated recommendations were systematically compared with the MDT decisions on a case-by-case basis. Three independent specialists, all blinded to the original MDT outcomes, evaluated each pair of decisions. Concordance for each case was defined according to the majority agreement among these blinded reviewers.

In the scoring system, ‘0’ indicates that no treatment or additional investigation is required, whereas ‘1’ indicates that treatment or further diagnostic work-up is warranted.

The paired binary output reflects the decisions of both parties using the following structure: the first digit represents the MDT decision, and the second digit represents the corresponding AI decision.

• 0–0: both MDT and AI agree that no treatment/investigation is needed (full concordance)
• 1–1: both MDT and AI agree that treatment/investigation is required (full concordance)
• 0–1 or 1–0: MDT and AI disagree; one recommends intervention while the other does not (discordance)

This system allows for a direct and transparent comparison of MDT and AI decisions for every patient.

### 2.3. Statistical Methods

All data analyses in this study were conducted using the R programming language (version 4.3.2). In the first stage, descriptive statistics were examined to characterize the demographic and clinical features of the patient cohort. Frequencies, percentages, means, and standard deviations were calculated for demographic variables. Continuous variables such as FVC and DLCO were dichotomized into “decreased” or “not decreased” categories based on the clinically relevant 80% threshold. Multilevel categorical variables, such as diagnostic groups, were consolidated into an “Other” category when appropriate, in order to avoid potential issues in logistic regression modeling. Variables were selected based on clinical relevance, prior literature, and data availability. Multicollinearity was assessed using variance inflation factors, and highly collinear variables were excluded from the final models to ensure model stability. In the second stage, agreement between AI-generated and MDT decisions was assessed using Cohen’s Kappa (κ) statistic where the κ (kappa) values represent the level of agreement: <0.20 = slight, 0.21–0.40 = fair, 0.41–0.60 = moderate, 0.61–0.80 = substantial, >0.80 = almost perfect agreement. These analyses were performed using the irr and psych packages in R. The κ coefficient was used as a chance-corrected measure of agreement. Subsequently, logistic regression analyses were conducted to identify predictors for each type of decision agreement (immunosuppressive treatment, follow-up without medication, antifibrotic treatment, further investigation required, clinical diagnosis agreement, and radiological diagnosis agreement). These models were fitted using the glm () function in R, specifying the binomial family. Statistical significance of the models was evaluated through *p*-values, while overall model fit was assessed using the Likelihood Ratio Test.

## 3. Results

An examination of the demographic characteristics of this cohort study (*n* = 47) revealed a predominance of female patients (61.70%, *n* = 29). The mean age was 61.74 ± 10.40 years. The most frequently observed diagnosis was RA, accounting for 31.91% of cases (*n* = 15). This was followed by cases of AAV, IPAF, and sarcoidosis. Nearly half of the patients (48.94%) had no comorbidities, while the proportion of current smokers remained relatively low at 23.40%. The results are summarized in Table 1, including clinical findings and test results. Strikingly, a reduction in DLCO was observed in nearly all patients (97.87%), with only one individual demonstrating values within the normal range.

The analyses indicate a statistically significant level of agreement across all decision types (*p* < 0.001), as shown in Table 2. For clinical diagnosis decisions, agreement was moderate (κ = 0.52), suggesting that the AI system can reach partially consistent conclusions in diagnostic processes, yet disagreements between categories remain a substantial challenge (34%, *n* = 16). In contrast, immunosuppressive treatment necessity and follow-up without medication decisions demonstrated a higher level of concordance, reaching the moderate-to-high range (κ = 0.64 and κ = 0.67, respectively). This pattern indicates that the AI tends to provide consistent binary (“yes/no”) treatment recommendations. Accordingly, discordance in these decision categories remained relatively low—for example, observed in only *n* = 8 and *n* = 7 patients, respectively—underscoring the AI’s close concordance with expert panel judgments in such contexts. Antifibrotic treatment decisions yielded moderate agreement (κ = 0.49), while radiological diagnoses showed a similar level of alignment (κ = 0.55), particularly in patterns such as UIP and fNSIP, each contributing 10.6% agreement. The lowest, yet still moderate, concordance was noted in decisions regarding further investigations (κ = 0.45), where the AI and MDT diverged almost equally (21.28% vs. 19.15%). Specifically, the MDT recommended additional testing in 10 cases that the AI did not, whereas the AI suggested further work-up in 9 cases the MDT deemed unnecessary—highlighting a meaningful degree of discordance (Table 2). Notably, when it came to actionable recommendations, the AI system consistently proposed a broader set of potential interventions compared to the MDT, reflecting a more expansive interpretive approach.

The distribution of agreement rates further illustrates the AI’s reliability. Almost one-third of patients (29.79%, *n* = 14) demonstrated concordance with the panel in five out of six decision domains, indicating that occasional discrepancies did not substantially undermine overall alignment. Moreover, complete agreement across all six categories was observed in 11 patients (23.40%), highlighting the AI’s potential to serve as a robust adjunct in multidisciplinary decision-making (Table 2).

Multivariable logistic regression did not reveal statistically significant predictors across the six decision domains, with overall model fit remaining non-significant (*p* = 0.083–0.828) (Table 3). In immunosuppressive treatment decisions, comorbidity burden showed a negative trend (β = −0.610, *p* = 0.090), suggesting reduced likelihood of therapy in patients with multiple comorbidities, while ANA positivity approached significance (β = 1.726, *p* = 0.060), indicating a possible increased propensity for treatment in this subgroup. No meaningful predictors emerged for antifibrotic therapy, follow-up without medication, or diagnostic concordance. Cough displayed a borderline association with further investigation (β = −1.384, *p* = 0.071), implying that its absence may prompt additional testing (Table 3). Collectively, these findings indicate that AI-panel agreement patterns are unlikely to be accounted for by standard demographic, clinical, or serological variables, underscoring the complexity of the decision-making process.

## 4. Discussion

Our study revealed a statistically significant level of agreement between the MDT and AI system across all decision types. In clinical and radiologic preliminary diagnoses, as well as in decisions regarding anti-inflammatory and antifibrotic therapy, drug-free follow-up, and additional diagnostic testing, at least a moderate level of concordance was observed. In a recent study, a rheumatology team and Chat-GPT-4 were each asked to provide first-line treatment recommendations for 20 hypothetical patients. While no significant differences were observed in terms of safety between the initial treatment plans, the rheumatologists achieved higher scores in guideline adherence, medical appropriateness, completeness, and overall quality. The large language model (LLM)-generated plans were notably longer and more detailed [17]. In another noteworthy study, 25 clinical questions encompassing five rheumatic diseases—SLE, RA, ankylosing spondylitis, psoriatic arthritis, and fibromyalgia—were developed. Responses were generated both by Chat-GPT-4.0 and by physicians with varying levels of rheumatology experience, allowing for direct comparison. Two blinded rheumatologists independently evaluated the answers. The highest overall performance was achieved by physicians with 5 to 10 years of clinical practice, followed closely by Chat-GPT-4, which reached a 68% agreement rate. Notably, Chat-GPT-4 attained 100% accuracy in domains concerning the selection of first-line therapeutic options. In contrast, its performance was weakest in recognizing the most informative clinical signs and symptoms—a task that often depends on nuanced clinical judgment [18]. In our study, the strongest agreement was also observed for treatment-related decisions, particularly regarding anti-inflammatory therapy and the appropriateness of drug-free follow-up. In contrast, the need for antifibrotic therapy achieved only moderate concordance. This discrepancy likely reflects the fact that anti-inflammatory strategies and watchful waiting are guided by well-established protocols and standardized clinical practice. Concerns about adverse effects of immunosuppressive agents—such as hypogammaglobulinemia and infection risk—may further influence therapeutic choices [19] whereas antifibrotic therapy remains relatively novel and less consistently integrated into rheumatic diseases. Moreover, antifibrotic indications rely heavily on accurate identification of fibrotic radiological patterns or evidence of progressive disease. Borderline or heterogeneous presentations can introduce interpretive variability, thereby reducing alignment between the AI system and expert panel compared with more straightforward treatment categories [20]. However, in our study, radiological concordance was achieved in roughly two-thirds of the patients, with the highest agreement observed in cases of UIP and fibrotic NSIP. In fact, the MDT and the AI produced identical diagnoses in all 10 patients with these patterns, underscoring the exceptionally high level of concordance. Consistent with our findings, a multicenter externally validated study showed that a deep learning model could differentiate UIP from non-UIP patterns with diagnostic and prognostic accuracy comparable to expert thoracic radiologists [21]. Taken as a whole, the strong alignment observed between AI-generated outputs and clinical assessments underscores the value of the system as an adjunctive decision-support tool. Its recommendations may meaningfully inform and refine clinical reasoning; however, they must remain subordinate to holistic clinical appraisal and multidisciplinary expertise, rather than serving as a substitute for them.

The lowest level of agreement between clinicians and the AI system concerned decisions regarding the need for additional diagnostic procedures. In approximately 60% of cases, both the multidisciplinary team (MDT) and ChatGPT concurred that no further testing was required. In contrast, in 10 patients (21%), the AI system advised supplementary investigations that the MDT considered unnecessary. Most discordant instances were driven by the AI’s inclination to recommend extended diagnostic evaluations. Specifically, the model frequently proposed broader assessments—such as bronchoscopy or follow-up imaging—particularly when radiological findings were atypical or functional parameters were borderline, whereas MDT decisions were informed by longitudinal disease stability and broader clinical context. This discrepancy underscores the model’s constrained ability to incorporate temporal clinical reasoning and nuanced risk–benefit evaluation. Notably, in cases where additional testing was clinically justified, the AI-generated recommendations were notably thorough and detailed, closely mirroring the patterns described by Labinsky et al. [17] This result may support the view that Chat-GPT has limitations in appreciating the clinical judgment and nuanced considerations, consistent with the findings of the aforementioned second study [18]. Its tendency to suggest more extensive diagnostic work-ups appears to stem from a deliberately cautious, safety-driven reasoning framework that favors thoroughness when clinical context is incomplete, rather than from inappropriate overuse. While this approach may help minimize the risk of overlooked pathology, it also highlights the system’s inability to fully integrate longitudinal clinical judgment, individualized risk–benefit assessments, and real-world resource considerations.

AI is rapidly reshaping rheumatology by offering fresh perspectives on how we diagnose and assess SARDs [22]. A simple, clinician-friendly machine learning–based model incorporating 14 clinical and serological features was developed, achieving a diagnostic accuracy of 94%. Although the ANA test does not determine treatment necessity or specific organ involvement, in that study, ANA positivity emerged as one of the principal variables classifying patients as SLE [23]. Given that ANA is frequently considered as a hallmark for diagnosing connective tissue diseases, several authors caution that over-reliance on this marker may contribute to misdiagnosis and unnecessary treatment exposure [24]. Within our cohort, ANA positivity, although not reaching statistical significance, appeared to be potentially associated with clinical decisions regarding the initiation of anti-inflammatory or immunosuppressive therapy rather than diagnostic determination. This pattern suggests that ANA positivity may have subtly informed therapeutic judgment, pointing to a possible—but not conclusive—association with a higher likelihood of initiating immunosuppressive treatment. Another observation of interest in our study concerned the presence of cough. In the guidelines, progressive dyspnea, persistent chronic cough, and exercise-induced hypoxemia are emphasized as critical red flags that should alert both patients and physicians to the need for further diagnostic evaluation and differential diagnosis. These clinical warning signs are regarded as the most decisive indicators prompting comprehensive assessment and early multidisciplinary discussion [25]. Although statistical significance was not reached, the presence of cough appeared to be associated with a potentially unfavorable clinical impact, warranting further exploration. This tendency may reflect its interpretation as a marker of concomitant pneumonia or as a clinical manifestation of primary disease involvement. Given the absence of statistical significance, these observations should be regarded as hypothesis-generating rather than confirmatory.

When interpreting our findings, it is essential to emphasize that AI should be positioned as an adjunct to, rather than a replacement for, multidisciplinary clinical judgment. While AI may enhance diagnostic reasoning, it showed a consistent inclination toward recommending broader investigations, more intensive interventions, and closer surveillance. If applied without critical appraisal, this tendency carries the risk of unnecessary testing, overdiagnosis, and inefficient use of healthcare resources. Accordingly, our results underscore the need for AI-generated outputs to be carefully contextualized and moderated by multidisciplinary expertise to maintain judicious, patient-centered decision-making. Moreover, the clinical deployment of AI extends beyond technical accuracy to encompass important ethical and medico-legal considerations, including responsibility for clinical decisions and the potential for overtreatment. These issues further affirm that AI should function strictly as a decision-support instrument, not as an independent clinical authority.

## 5. Limitations

The heterogeneity of the underlying rheumatic diseases represents a key limitation, further amplified by the single-center design of the cohort. MDT decisions were used as the reference standard, thereby implicitly presuming their validity and ethical soundness, despite the absence of a formal assessment of guideline adherence for either MDT- or AI-based recommendations. Although assigning acceptability scores to individual outputs might have provided more detailed discrimination, concordance was instead established through consensus among three independent, blinded reviewers. Due to the exploratory nature of the study, no a priori power calculation was performed; all consecutively eligible patients were included, and the relatively small sample size (*n* = 47) limited the ability to identify modest effects. Accordingly, statistical inferences should be interpreted cautiously, and confirmation in larger, multicenter cohorts with more homogeneous disease spectra and prospectively defined power assumptions is warranted. Finally, center-specific practice patterns may shape MDT deliberations, thereby constraining the generalizability of these findings to institutions with differing organizational models or clinical cultures.

## 6. Conclusions

In patients with SARDs and pulmonary involvement—especially in diagnostically challenging cases—we observed meaningful concordance between MDT decisions and AI-generated recommendations across key domains, including diagnostic prioritization, treatment selection, suitability for drug-free follow-up, and the need for further investigations. Despite this alignment, AI should not be interpreted as a replacement for multidisciplinary expertise. Its role is inherently auxiliary, and its outputs must be interpreted through careful clinical judgment. Notably, AI showed a consistent tendency to suggest broader testing and more aggressive interventions. Without critical evaluation, such patterns could lead to unnecessary investigations, overdiagnosis, and the inefficient use of healthcare resources. These findings underscore a central clinical warning: AI recommendations should always be contextualized and validated within an MDT framework to ensure safe, balanced, and patient-centered care.

## Figures and Tables

**Table 1:** Diagnosis, Clinical Findings, Laboratory and Pulmonary Function Test Characteristics of the Study Population.

Variable	Category	*n* (%) or Mean ± SD
**Gender**	Male	18 (38.30%)
	Female	29 (61.70%)
**Age (years)**		61.74 ± 10.40
**Disease duration (months)**		45.80 ± 8.62
**Diagnosis**	Rheumatoid arthritis	15 (31.91%)
	ANCA-associated vasculitis	6 (12.77%)
	Interstitial pneumonia with autoimmune features	6 (12.77%)
	Sarcoidosis	6 (12.77%)
	Sjögren’s syndrome	3 (6.38%)
	Mixed connective tissue disease	2 (4.26%)
	Systemic sclerosis	2 (4.26%)
	Others	7 (14.89%)
**Number of comorbidities**	0	23 (48.94%)
	1	11 (23.40%)
	2	9 (19.15%)
	3	2 (4.26%)
	4	2 (4.26%)
**Smoking status**	Current smoker	11 (23.40%)
	Non-smoker	36 (76.60%)
**Dyspnea**	Present	19 (40.43%)
	Absent	28 (59.57%)
**Cough**	Present	19 (40.43%)
	Absent	28 (59.57%)
**Fever**	Present	5 (10.64%)
	Absent	42 (89.36%)
**Rheumatoid factor**	Positive	17 (36.17%)
	Negative	30 (63.83%)
**Anti-CCP antibody**	Positive	12 (25.53%)
	Negative	35 (74.47%)
**ANA**	Positive	22 (46.81%)
	Negative	25 (53.19%)
**ANCA**	Positive	8 (17.02%)
	Negative	39 (82.98%)
**Serum ACE**	Elevated	6 (12.77%)
	Normal	41 (87.23%)
**Ongoing steroid use**	Yes	16 (34.04%)
	No	31 (65.96%)
**CRP (mg/L)**		26.83 ± 58.51
**Urea (mg/dL)**		39.43 ± 20.60
**Creatinine (mg/dL)**		0.63 ± 0.45
**AST (U/L)**		18.70 ± 9.26
**ALT (U/L)**		17.89 ± 8.74
**WBC (/mm^3^)**		8755.11 ± 3192.18
**Hemoglobin (g/dL)**		12.75 ± 1.95
**Platelets (/mm^3^)**		286,042.55 ± 114,507.82
**FEV_1_ (% predicted)**		55.19 ± 37.97
	Low	33 (70.21%)
	Normal	14 (29.79%)
**FVC (% predicted)**		57.02 ± 38.83
	Low	33 (70.21%)
	Normal	14 (29.79%)
**DLCO (% predicted)**		36.19 ± 31.31
	Low	46 (97.87%)
	Normal	1 (2.13%)

Abbreviations: ANCA—Antineutrophil cytoplasmic antibody; CCP—Cyclic citrullinated peptide antibody; ANA—Antinuclear antibody; ACE—Angiotensin-converting enzyme; CRP—C-reactive protein; AST—Aspartate aminotransferase; ALT—Alanine aminotransferase; WBC—White blood cell count; FEV_1_—Forced expiratory volume in one second; FVC—Forced vital capacity; DLCO—Diffusing capacity of the lungs for carbon monoxide.

**Table 2:** Agreement Levels Between MDT Decisions and AI-Based Recommendations Across Clinical Categories.

The Questions	Subcategory	*n* (%) or κ, Agreement Level
What is the primary clinical differential diagnosis?	Rheumatoid arthritis with ILD	8 (17.02%)
	ANCA vasculitis with pulmonary involvement	5 (10.64%)
	Inactive sarcoidosis	3 (6.38%)
	IPAF with ILD	2 (4.26%)
	Sjögren’s syndrome with ILD	2 (4.26%)
	Other disease agreements	11 (23.40%)
	Disagreement	16 (34.04%)
	**Overall agreement**	**κ = 0.52, Moderate**
What is the primary radiologic differential diagnosis?	UIP	5 (10.64%)
	fNSIP	5 (10.64%)
	AAV with pulmonary involvement (DAH)	2 (4.26%)
	Cavitating nodule	2 (4.26%)
	Rheumatoid nodule	2 (4.26%)
	Other agreements (OP, LIP, drug reactions)	14 (29.79%)
	Disagreement	17 (36.17%)
	**Overall agreement**	**κ = 0.55, Moderate**
Is anti-inflammatory immunosuppressive therapy indicated? **(0: no, 1: yes)**	Agreement (0–0)	14 (29.79%)
	Agreement (1–1)	25 (53.19%)
	Disagreement (0–1)	2 (4.26%)
	Disagreement (1–0)	6 (12.77%)
	**Overall agreement**	**κ = 0.64, Moderate–High**
Is antifibrotic therapy indicated? **(0: no, 1: yes)**	Agreement (0–0)	39 (82.98%)
	Agreement (1–1)	3 (6.38%)
	Disagreement (0–1)	4 (8.51%)
	Disagreement (1–0)	1 (2.13%)
	**Overall agreement**	**κ = 0.49, Moderate**
Is drug-free follow-up appropriate? **(0: no, 1: yes)**	Agreement (0–0)	27 (57.45%)
	Agreement (1–1)	13 (27.66%)
	Disagreement (0–1)	4 (8.51%)
	Disagreement (1–0)	3 (6.38%)
	**Overall agreement**	**κ = 0.67, Moderate–High**
Is additional diagnostic testing indicated? **(0: no, 1: yes)**	Agreement (0–0)	28 (59.57%)
	Agreement (1–1)	0 (0.00%)
	Disagreement (0–1)	10 (21.28%)
	Disagreement (1–0)	9 (19.15%)
	**Overall agreement**	**κ = 0.45, Moderate**
Overall Agreement Distribution on Each Decision Domains	1	1 (2.13%)
	2	4 (8.51%)
	3	5 (10.64%)
	4	12 (25.53%)
	5	**14 (29.79%)**
	6	**11 (23.40%)**

Abbreviations: ILD—Interstitial Lung Disease; ANCA—Antineutrophil cytoplasmic antibody; IPAF—Interstitial Pneumonia with Autoimmune Features; UIP—Usual Interstitial Pneumonia; fNSIP—Fibrotic Nonspecific Interstitial Pneumonia. AAV—Antineutrophil cytoplasmic antibody–associated vasculitis; DAH—Diffuse alveolar hemorrhage; OP—Organizing pneumonia; LIP—Lymphoid interstitial pneumonia. Note: 0 = no treatment/investigation needed, 1 = treatment/investigation indicated. Agreement (0–0) indicates concordance on no treatment/investigation, Agreement (1–1) indicates concordance on treatment/investigation necessity. Disagreement (0–1) or (1–0) indicates discordance between MDT decisions and AI recommendations. The paired binary scores (e.g., 0–0, 0–1, 1–0, 1–1) represent the decision of the MDT decision (first digit) and the corresponding decision generated by the artificial intelligence model (second digit). κ (kappa) values represent the level of agreement: <0.20 = slight, 0.21–0.40 = fair, 0.41–0.60 = moderate, 0.61–0.80 = substantial, >0.80 = almost perfect agreement.

**Table 3:** Multivariable Logistic Regression Analysis for Predictors Across Decision Categories.

Predictor	Immunosuppressive Treatment	Antifibrotic Treatment	Follow-Up Without Medication	Further Investigation Required	Clinical Diagnosis Agreement	Radiological Diagnosis Agreement
**Diagnosis**	0.062 (0.980)	−8.897 (0.133)	−1.303 (0.515)	0.276 (0.570)	271.512 (0.999)	−135.216 (0.999)
**Age**	−0.002 (0.954)	0.099 (0.254)	0.001 (0.961)	-	−3.943 (0.999)	3.846 (0.999)
**Smoking**	-	−2.199 (0.996)	-	−0.937 (0.240)	-	46.438 (1.000)
**Number of comorbidities**	−0.610 (0.090) *	-	MC	-	-	-
**ESR**	-	-	0.017 (0.445)	-	-	-
**CRP**	-	-	−0.005 (0.563)	-	-	-
**Dyspnea**	-	-	-	−0.353 (0.622)	-	-
**Cough**	-	-	-	−1.384 (0.071) *	-	-
**Fever**	-	-	-	1.764 (0.123)	-	-
**FEV_1_ (no decrease)**	2.426 (0.206)	−20.703 (1.000)	0.105 (0.948)	-	-	−19.805 (1.000)
**FVC (no decrease)**	−2.263 (0.256)	21.897 (1.000)	−0.123 (0.938)	-	-	−27.742 (1.000)
**DLCO (no decrease)**	−51.857 (1.000)	−19.347 (0.999)	17.638 (0.994)	-	-	2.413 (1.000)
**Current treatment**	MC	MC	MC	MC	MC	MC
**Ongoing Steroid therapy (yes)**	0.666 (0.444)	-	-	-	-	-
**RF**	0.514 (0.681)	-	-	-	97.075 (0.999)	66.701 (1.000)
**CCP**	0.171 (0.907)	-	-	-	−41.449 (1.000)	−114.172 (1.000)
**ANA**	1.726 (0.060) *	-	-	-	48.454 (0.999)	47.875 (0.999)
**ANCA**	1.091 (0.323)	-	-	-	−60.243 (0.999)	−97.385 (1.000)
**Serum ACE**	-	-	-	-	−33.673 (1.000)	−4.633 (1.000)
**Model significance (** ***p*** **)**	0.437	0.273	0.828	0.159	0.288	0.083

Abbreviations: MC—Multicollinearity (variable excluded from the model due to high collinearity); ESR—Erythrocyte sedimentation rate; CRP—C-reactive protein; FEV_1_—Forced expiratory volume in one second; FVC—Forced vital capacity; DLCO—Diffusing capacity of the lungs for carbon monoxide; RF—Rheumatoid factor; CCP—Cyclic citrullinated peptide antibody; ANA—Antinuclear antibody; ANCA—Antineutrophil cytoplasmic antibody; ACE—Angiotensin-converting enzyme. * lacks statistical significance but retains potential relevance from a statistical perspective.

## Data Availability

The data presented in this study are available on request from the corresponding author due to ethical and privacy considerations, as they contain sensitive patient-level clinical information, and therefore are not publicly available. De-identified data may be provided upon reasonable request, subject to institutional regulations and approval by the local ethics committee.
